# Assessing the Supportiveness of Healthcare Environments’ Light and Color: Development and Validation of the Light and Color Questionnaire (LCQ)

**DOI:** 10.1177/1937586720975209

**Published:** 2020-11-28

**Authors:** Jeanette Lindahl, Hans Thulesius, Mikael Rask, Helle Wijk, David Edvardsson, Carina Elmqvist

**Affiliations:** 1 Centre for Interprofessional Cooperation Within Emergency Care (CICE), Department of Health and Caring Sciences, 249958 Linnaeus University, Växjö, Sweden; 2 Department of Research and Development, Region Kronoberg, Växjö, Sweden; 3 Family Medicine, Department of Clinical Sciences, 5193 Lund University, Malmö, Sweden; 4 Faculty of Health and Life Sciences, Department of Medicine and Optometry, 5193 Linnaeus University, Växjö, Sweden; 5 Department of Health and Caring Sciences, 5193 Linnaeus University, Växjö, Sweden; 6 Institute of Health and Care Sciences, Sahlgrenska Academy, 3570 University of Gothenburg, Sweden; 7 School of Nursing and Midwifery, La Trobe University, Melbourne, Victoria, Australia; 8 Department of Nursing, 211742 Umeå University, Sweden

**Keywords:** color, emergency department, family members, instrument development, light, patients, physical care environment, psychometrics, self-reported questionnaire, staff

## Abstract

**Aim:**

The aim of this study was to develop and evaluate a self-report instrument measuring patients’, family members’, and staff’s perceived support from light and color in the physical environment of an emergency department (ED)—the Light and Color Questionnaire (LCQ).

**Background:**

The physical care environment is an important part of a comprehensive caring approach in all levels of care not only for patients but also for family members and staff. However, no existing self-report questionnaire assessing the extent to which light and color are perceived as being supportive in the physical care environment from the users’ perspective was found.

**Method:**

The LCQ was developed as part of a pre–post study in which an ED serving 125,000 people was refurbished and remodeled using evidence-based design. The LCQ consists of six items for light and five items for color and assesses awareness/orientation, safety/security, functional abilities, privacy, personal control, and stimulation. The study was carried out in four steps: constructions of items, assessment of face validity, data collection, and data analysis.

**Result/Conclusion:**

Psychometric evaluation of the two versions, LCQ-Patient/Family member and LCQ-Staff, showed satisfactory content and internal validity (>90%) and high internal consistency (Cronbach’s coefficient α = .9) to support the use of the questionnaire for research and development purposes. Explorative factor analysis of a total of 600 questionnaire responses confirmed light and color as distinctive and independent dimensions creating perceptions of more or less supportiveness for respondents. The LCQ instrument may be useful for architects, administrators, and researchers of healthcare environments.

## Background

The physical care environment has been described as an important and complex part of a comprehensive caring approach (Caspari et al., 2011; Ulrich et al., 2008) not only for patients but also for family members and staff (Gerhardsson et al., 2020; Gesler et al., 2004; Huisman et al., 2012; Mahmood & Tayib, 2019). Light provides important information as a link between humans and their surroundings, and it creates our visual experience of the world together with color (Klarén, 2017; Laike, 2017). To understand and perceive a room or situation, it is necessary for light and color to be exposed at the same since they, are in context, always affect each other (Klarén, 2017). The electromagnetic radiation that contributes to light and color can be measured with instruments and thereby be described by intensity or wavelength ranges of light radiation at a given point in the room. However, color and light experiences, as for example, light radiation intensity, variety, and spectral distribution, can have an effect on the human organism in terms of alertness, well-being, and behavior (Klarén, 2017). Light and color are defined as individual perceptions and subjective visual sensations linked to physical, physiological, cultural, and social reactions, uniquely affecting the five senses of each individual (Klarén, 2017; Mahnke, 1996).

***The physical care environment has been described as an important and complex part of a comprehensive caring approach***.

***. . . not only for patients but also for family members and staff***

***Light and color are defined as individual perceptions and subjective visual sensations linked to physical, physiological, cultural, and social reactions, uniquely affecting the five senses of each individual***

Without light, there are no colors, and without color, there are no contrasts or the ability to visually perceive a room. Furthermore, light is never constant since daylight varies (Anter & Klarén, 2017). Daylight and artificial light affect humans in many different ways (Benedetti et al., 2001; Gerhardsson et al., 2020; Laike, 2017), in the short term, light affects our alertness, sleep, circadian regulation, mood, and accomplishment, and in the long term, it affects our health and well-being (Gerhardsson et al., 2020; Laike, 2017). Therefore, light is considered an important environmental factor (Gharaveis & Kazem-Zadeh, 2018; Gharaveis et al., 2016; Ulrich et al., 2008).

A simply understood physical care environment that is easy to interpret has been described when promoting and supporting safety, independence, and well-being in a stressful situation (Davis & Weisbeck, 2016; Mahnke, 1996; Wijk, 2001), in terms of orientation, control, and space for social support and distraction (Wijk & Häggström, 2017).

It has been argued that a supportive physical care environment can benefit especially older persons regardless of whether they are patients, family members, or staff (Joseph et al., 2015; Reiling et al., 2008). Appropriate lighting and colors, both natural and artificial, along with access to natural daylight through windows, have been described as crucial to support all users (Joseph et al., 2015; Salonen et al., 2013) and to improve opportunities to orientate and locate (Hidayetoglu et al., 2012; Salonen et al., 2013; Ulrich et al., 2008; Williams et al., 2008). In contrast, suboptimal light and color conditions have been described as contributing to medication errors, worsening staff performance (Salonen et al., 2013), fall injures (Hignett & Masud, 2006), longer hospital stays (Ulrich, 1984, 2012; Ulrich et al., 2008), and lowering the quality of life (Garre-Olmo et al., 2012). Although it has been acknowledged that the physical environment at hospitals has a significant impact on health and safety, its construction is seldom acknowledged as a means of enhancing patient safety (Reiling et al., 2008). However, the facility design of hospitals increasingly emphasizes innovations in lighting levels and color (Dalke et al., 2006; Reiling et al., 2008) to create supportive physical care environments that help individuals reach their optimal level of functioning (Innes & McCabe, 2007; Joseph et al., 2015; Wijk, 2001; Wijk & Häggström, 2017). According to evidence-based design (Ulrich, 2012), the built environment should be based on the best available research. However, knowledge of how light and color in physical care environments are perceived to support users is scarce.

***Although it has been acknowledged that the physical environment at hospitals has a significant impact on health and safety, its construction is seldom acknowledged as a means of enhancing patient safety***.

***According to evidence-based design***

***. . . the built environment should be based on the best available research***

In the study of physical care environment, the Professional Environmental Assessment Protocol (PEAP; Lawton et al., 2000; Norris-Baker et al., 1999; Weisman, 1994) appears to be the most commonly used and seems to be the best validated questionnaire focused on the extent to which the physical setting supports users in special care units and people in nursing homes (Elf et al., 2017). However, an important piece of information is missing since the PEAP is intended for care managers without including experiences from the users’ perspective. No self-report instruments assessing the extent to which the physical care environment or light and color are perceived as being supportive from the users’ perspective have to our knowledge been published remove and add (a.a). This instrument should contribute to the literature by providing means to assess the extent to which the light and color of healthcare environment are perceived by users as being supportive to them. Therefore, the aim of this study was to develop and evaluate a self-report questionnaire measuring patients’, family members’, and staff’s perceived support from light and color in the physical environment of an emergency department (ED)—the Light and Color Questionnaire (LCQ).

## Method

### Design and Setting

This instrument development study was conducted in four steps: (1) Construction of items, (2) assessment on face validity, (3) Participants and data collection, and (4) data analysis. The questionnaire was designed to evaluate whether patients, family members, and staff perceived support from light and color in the physical care environment of an ED serving 125,000 people in southern Sweden. The questionnaire was a part of a nonrandomized trial with pre- and posttesting in the Caring Optimized Physical Environment (COPE) study, in which an ED was being refurbished and remodeled according to an evidence-based design (Ulrich et al., 2008). The COPE study was a collaboration between an expert group, including two assistant nurses, one registered nurse, and three nurse managers from the ED, and a research team (including the first and last author and a senior lecturer), as well as three architects. This approach resulted in a redesigned ED with more natural light and windows, including windows on the inner walls and doors, along with the opportunity to choose light settings in the rooms. A solid color for all floors was chosen in rooms intended for patients and family members. Wall color was selected based on the function of the rooms. Other intervention details and results from the COPE study will be presented elsewhere.

### Development of the LCQ

#### Step 1: Construction of items

The LCQ was constructed based on PEAP’s eight dimensions of the physical care environment: maximizing safety and security, maximizing awareness and orientation, supporting functional abilities, facilitating social contact, providing privacy, opportunities for personal control, regulation and quality of stimulation, and continuity of self. The LCQ was developed by the research team and an expert group. The questionnaire development started with a translation and back translation of the PEAP protocol (Weisman, 1994). Minor discrepancies in translation were resolved by consensus discussions in the research team.

The need for support from the physical environment can differ somewhat between patients, family members, and staff. For patients and family members, there are support needs from the perspective of being cared for in an unfamiliar environment. For example, light and color could enhance personal control by making it easier to orientate. For staff members, there are support needs from the perspective of working in a well-known environment and performing functional tasks, where support from light and color helps provide a safer and more secure working environment, which also improves patient safety. Therefore, two questionnaires were created: one for patients and family members (LCQ-Patient/Family [LCQ-P/F]) and one for staff members (LCQ-Staff [LCQ-S]). Then, the research team separately formulated questions for light and color, based on the eight PEAP domains, except for *opportunity for personal control* (excluded in the items for color) and *facilitation of social contact* and *continuity of self* (excluded for both light and color), since these were considered irrelevant in the ED context. The questionnaires resulted in six domains for light and five domains for color with 37 preliminary items in total.

***The need for support from the physical environment can differ somewhat between patients, family members, and staff.***

***. . . two questionnaires were created: one for patients and family members (LCQPatient/Family [LCQ-P/F]) and one for staff members (LCQ-Staff [LCQ-S]).***

#### Step 2: Assessment of face validity

After the construction of the first drafts of the questionnaires, a content face validity check was performed in a seminar by the research team and the expert group. During the seminar, all items were discussed, and after consensus decisions, the items were reduced to one for each of the selected main domains of the PEAP since there were similar items in each PEAP main domain. This resulted in six items for light and five items for color, for a total of 11 items on each subset; the LCQ-P/F and LCQ-S were similar except for different perspectives of patients, family members, and staff.

An ED staff group (assistant nurses, registered nurses, and physicians) piloted the 11-item LCQ-P/F and LCQ-S, and members of the expert group distributed and collected the questionnaires for the pilot survey. The content face validity check resulted in adjustments for background questions and linguistic adjustments. All 11 items were formulated as statements built on a 6-point Likert-type scale (0 = *no, I disagree completely* to 5 = *yes, I agree completely*). Maximum score for each item was thus 5. The questionnaire also allowed space for open-ended comments. The LCQ-P/F and LCQ-S Swedish versions were translated into English and then back translated into Swedish by two Swedish-speaking postdoc researchers fluent in English. There were only minor differences between the two translators’ versions, and consensus was reached within the research team (Tables 1 and 2).

**Table 1.** Light and Color Questionnaire for Patients/Family members (LCQ-P/F).

Professional Environmental Assessment Protocol	The Place Where I am Now Being Cared for Has a Lighting That	The Place Where I am Now Being Cared for Has a Coloring That
Maximize awareness and orientation	Item 1. Helps me to find my way and orient myself.	Item 7. Helps me to find my way and orient myself.
Maximize safety and security	Item 2. Helps me to feel safe and secure.	Item 8. Helps me to feel safe and secure.
Support functional abilities	Item 3. Helps me so that I can move like I am used to.	Item 9. Helps me so that I can move like I am used to.
Provision of privacy	Item 4. Helps me to feel private.	Item 10. Helps me to feel private.
Opportunities for personal control	Item 5. Helps me to be in control and have choices for my needs.	
Regulation and quality of stimulation	Item 6. Gives me the opportunity to get the right light for my needs.	Item 11. Affects my visit positively.

**Table 2.** Light and Color Questionnaire for Staff (LCQ-S).

Professional Environmental Assessment Protocol	The Place Where I am Working Has a Lighting That	The Place Where I am Working Has a Coloring That
Maximize awareness and orientation	Item 1. Helps me to find my way and orient myself.	Item 7. Helps me to find my way and orient myself.
Maximize safety and security	Item 2. Helps me to feel safe and secure for my work tasks.	Item 8. Helps me to feel safe and secure for my work tasks.
Support functional abilities	Item 3. Helps me to perform my work tasks as good as possible and without difficulties.	Item 9. Helps me to perform my work tasks as good as possible and without difficulties.
Provision of privacy	Item 4. Helps me to create integrity/privacy for the patient.	Item 10. Helps me to create integrity/privacy for the patient.
Opportunities for personal control	Item 5. Helps me to be in control and have choices in my work.	
Regulation and quality of stimulation	Item 6. Gives me the opportunity to get the right light for my work tasks.	Item 11. Affects my job positively.

#### Step 3: Participants and data collection

Participants in the questionnaire survey consisted of patients, family members, and staff before and after the intervention (i.e., refurbishing and remodeling the ED). For each of the three groups, 100 survey responses were collected before and after the intervention. The total number of survey participants was thus 600. Psychometric sample size estimations recommend having samples of between five and 15 participants per item of the questionnaire being evaluated (Nunnally, 1978). This means that 11 items would require at least 165 participants per subset of the LCQ instrument.

Inclusion criteria for patients and family members were as follows: They should have the ability to master written Swedish, they should be of age >18 years, and they should have the ability to personally answer the questionnaire. Patients who arrived by ambulance and were triaged for assessment within 30 min were excluded from the study since their conditions were considered too severe for relevant participation. After being triaged, patients and family members meeting the inclusion criteria received written information describing the study’s aim, procedures, and how their data would be managed. Inclusion criteria for staff were working at the ED as a physician, registered nurse, assistant nurse, or nurse student in clinical training >5 weeks (Table 3). The staff received oral and written information from the first author at a regular weekly meeting. All participants were informed about their right to withdraw from the study at any time and that a returned questionnaire implied written consent. Participants were asked to provide demographic information regarding their age and gender. Patients and family members indicated the type of clinic and whether they had visited the ED previously. Family members also marked the accompanying patient’s age. The staff were asked about their profession and work experience in the ED, as well as elsewhere in the healthcare sector. Patients and family members arriving at the ED between 8 a.m. and 8 p.m. received questionnaires and completed them during their ED visit. The staff received the questionnaires from the first author and completed them during work hours. All participants placed the completed questionnaire in a sealed box at the ED, which was emptied daily by the first author. Data collection ended after 100 questionnaires per group, and incident had been returned; in total, 600 questionnaires were completed (300 before and 300 after the refurbishing and remodeling).

**Table 3.** Sociodemographic and Clinical Characteristics of Participants Divided Into Patients, Family Members, and Staff.

Participant Characteristics	Patients *n* = 200 100 + 100	Family Members *n* = 200 100 + 100	Staff *n* = 200 100 + 100	Total *n* = 600 300 + 300
Age-group ^a^, median	46–55	46–55	46–55	46–55
Women/men, *n* (%)	105/95 (52/48)	132/68 (66/34)	142/58 (71/29)	379/221 (64/36)
Earlier ED visits, *n* (%)	159 (80)	176 (88)		
ED unit, *n* (%)				
Medicine	82 (41)	83 (42)		
Surgery	41 (21)	42 (21)		
Orthopedics	39 (20)	37 (18)		
Other	25 (12)	35 (17)		
Missing	13 (6)	3 (2)		
Profession, *n* (%)				
Registered nurse			97 (48)	
Assistant nurse			50 (25)	
Physician			36 (18)	
Students			14 (7)	
Missing			3 (2)	
Work experience >3 years *n* (%)			169 (84)	
ED experience >3 years *n* (%)			124 (62)	

*Note.* ED = emergency department.

^a^ Age groups = 18*–*25, 26*–*35, 36*–*45, 46*–*55, 56*–*65, 66*–*75, and >75.

#### Step 4: Data analysis

Statistical analysis tests for construct validity and internal consistency reliability were conducted. All data were analyzed using SPSS Statistics Version 21.0 (SPSS Inc. Chicago, IL).

### Construct Validity

The LCQ-P/F and the LCQ-S were both assessed using basic item performance tests: means, standard deviations (*SD*), standard errors (*SE*), variance, skewness, range, and percentile distribution. Exploratory factor analysis (Buffoli et al., 2016) was performed to determine the two LCQ versions’ construct validity: Inter-item correlation matrix for factoring appropriateness, Bartlett’s test of sphericity (*p* < .05), and Kaiser–Meyer–Olkin (KMO) values (>.8) were considered good enough (Pett et al., 2003; Watson, 2004). All KMO values >.4 for both versions were used as correlation matrix determinants. Exploratory factor analysis was done using principal component analysis with varimax rotation in order to underline clear factors and item construct validity (Pett et al., 2003; Watson, 2004). All factors with an eigenvalue >1 and a factor loading >.5 were retained. Factor loadings ≥.5 were considered practically significant (Hair et al., 1995). Descriptive statistics such as frequencies and cross-tabulations were used as complements to the exploratory factor analysis.

### Internal Consistency Reliability

The internal consistency was tested by calculation of Cronbach’s coefficient α and item-total correlations. The following cutoff scores for acceptable psychometric performance were used: Item performance was deemed satisfactory through normal distribution of scores, and internal consistency was satisfactory with a Cronbach’s coefficient α of >.7 and item-total correlations between .3 and .8 (Rattray & Jones, 2007). Ethical considerations according to the Helsinki Declaration (World Medical Association, 2013) were followed regarding risks/benefits, voluntariness, informed consent, and confidentiality. According to Swedish legislation, at the time of the study, an application to the Ethical Review Authority was not required. However, the study was reviewed and considered suitable by Region Kronoberg Research Ethics Council (9/2009)

## Results

### Construct Validity

The distribution of scores on the LCQ-P/F and LCQ-S indicated a normal distribution. The mean total score, *SD*, *SE*, variance, skewness, range, and percentiles for light and color and groups LCQ-P/F and LCQ-S are described in Table 4. Total score for LCQ-P/F and LCQ-S Items 1–6 (light) can range from minimum 0 to maximum 30, and for LCQ-P/F and LCQ-S Items 7–11 (color) can range from 0 to maximum 25. Higher scores indicate better perceptions of support. The mean values for individual items on the LCQ-P/F range from 3.49 to 4.26 for Items 1–6 (light) and 3.27 to 3.75 for Items 7–11 (color). The LCQ-S mean values range from 3.06 to 3.89 for Items 1–6 (light) and 2.42 to 2.78 for Items 7–11 (color). The total score for Items 1–6 (light) was higher than the total score for Items 7–11 (color) for both versions of LCQ-P/F and LCQ-S.

**Table 4.** Descriptive Statistics Regarding Participant Distribution on Total Scale Score and Subscale Scores With Regard to the Light and Color Questionnaire for Patients/Family members and Staff (LCQ-P/F and LCQ-S).

Statistical Details	Items 1–6 Light		Items 7–11 Color	
	Patients/Family Members *n* = 371	Staff *n* = 191	Patients/Family Members *n* = 371	Staff *n* = 191
Median	25	22	19	14
Mean	24.061	21.66	17.78	13.28
*SD*	5.52	6.00	6.11	6.78
*SE*	.29	.43	.32	.49
Variance	30.46	35.98	37.31	46.00
Skewness	−1.31	−0.68	−0.97	−0.26
Kurtosis	2.37	0.18	0.49	0.73
Range	30	28	25	25
Percentiles				
25th	21	18	15	9
50th	25	22	19	14
75th	28	26	22	19

*Note*. *SD* = standard deviation. *SE* = standard error.

Content face validity of the LCQ-P/F and LCQ-S was estimated as satisfactory by the expert group and is supported by the theoretical foundations of PEAP. The internal validity of the two versions showed a high score since all items had an internal response rate of >90%. The two versions’ construct validity was estimated using exploratory factor analysis, along with theoretical underpinnings and item development by Weisman (1994). Bartlett’s test of sphericity (LCQ-P/F = 3571.3 *p* < 0.001 and LCQ-S = 1892.5 *p* < 0.001) and KMO (LCQ-P/F = 0.9 and LCQ-S = 0.9) as measures of sampling adequacy and factor analysis models showed appropriate figures. Items on the LCQ-P/F and LCQ-S revealed a two-factor solution for both versions, based on eigenvalues >1 and visual inspection of the Scree plot. All 11 items showed appropriate communalities, LCQ-P/F ≥ 0.562 and LCQ-S ≥ 0.648. Factor analyses of the two versions of the LCQ do not reveal any double factor loadings >.5.

### Exploratory Factor Analysis of LCQ-P/F

As shown in Table 5, the two factors extracted from LCQ-P/F revealed a cumulative explained variance of 74.3%. The first factor *color as support* of the LCQ-P/F includes six items (Items 4, 7–11) and accounted for 63.6% of the variance, while the second factor, *light as support*, includes five items (Items 1–3, 5, and 6) and accounted for 10.7% of the variance. Factor 1, color as support (Items 4, 7–11), explained most of the variance in the analysis demonstrating that color was the strongest factor for patients and family members. In Factor 1, color as support, the core item was Item 10, *helps me to feel private*, as this factor displayed the highest factor loading score (.871). In Factor 2, light as support, the core item was Item 1, *helps me to find my way and orient myself* (.845). Item 4 (light), *helps me to feel private*, showed a discrepancy in the basic concept since it had its highest factor loading (.617) in the first factor (color as support).

***Factor 1, color as support (Items 4, 7–11), explained most of the variance in the analysis demonstrating that color was the strongest factor for patients and family members.***

**Table 5.** Explorative Factor Analysis (Varimax Rotated) of the Light and Color Questionnaire (LCQ) With Reference to Patients/Family Members (LCQ-P/F).

Items in Factor	P/F	P	F	P/F	P	F
	Factor 1 Color as Support			Factor 2 Light as Support		
	(*n* = 371)	(*n* = 183)	(*n* = 188)	(*n* = 371)	(*n* = 183)	(*n* = 188)
The place where I am now being cared for has a						
lighting that…(1–6)						
Item 1. Helps me to find my way and orient myself.	.235	.173	.288	.845	.905	.790
Item 2. Helps me to feel safe and secure.	.344	.307	.364	.801	.835	.779
Item 3. Helps me so that I can move like I am used to.	.289	.367	.201	.795	.762	.828
Item 4. Helps me feel private.	.617	.749	.496	.425	.293	.536
Item 5. Helps me to be in control and have choices for my needs.	.389	.512	.293	.775	.668	.844
Item 6. Gives me the opportunity to get the right light for my needs.	.384	.445	.325	.704	.641	.761
coloring that…(7–11)						
Item 7. Helps me to find my way and orient myself.	.808	.716	.855	.361	.480	.251
Item 8. Helps me to feel safe and secure.	.842	.774	.888	.359	.485	.268
Item 9. Helps me so that I can move like I used to.	.770	.714	.791	.447	.516	.426
Item 10. Helps me feel private.	.871	.916	.804	.237	.177	.326
Item 11. Affects my visit positively.	.820	.812	.801	.270	.290	.286
Eigenvalue	7.0	7.2	6.8	1.2	1.1	1.3
% of variance explained, %	63.6	65.8	61.6	10.7	10.1	12.2

*Note.* Cumulative percentage of variance explained = 74.3% (P = 75.9%; F = 73.8%). Cronbach’s coefficient α = .94 (P = .94; F = .94).

### Exploratory Factor Analysis of LCQ-S

The two factors extracted from LCQ-S (Table 6) revealed a cumulative explained variance of 76.3%. The first factor, light as support, of the LCQ-S includes six items (Items 1–6) and accounted for 59.2% of the variance, while the second factor, color as support, includes five items (Items 7–11) and accounted for 17.1% of the variance. Factor 1, light as support (Items 1–6), explained most of the variance in the analysis, demonstrating that light was the strongest and most obvious factor for the staff. In Factor 1, light as support, the core item was Item 2, *helps me to feel safe and secure*, as this factor displayed the highest factor loading score (.861). In Factor 2, color as support, the core item was Item 9, *helps me to perform my work tasks as well as possible and without difficulties* (.919).

***Factor 1, light as support (Items 1–6), explained most of the variance in the analysis, demonstrating that light was the strongest and most obvious factor for the staff.***

**Table 6.** Explorative Factor Analysis (Varimax Rotated) of the Light and Color Questionnaire (LCQ) With Reference to Staff (LCQ-S).

Items in Factor	S	S	
	Factor 1 Light as Support (*n* = 191)	Factor 2 Color as Support (*n* = 190)	
The place where I am working has a			
lighting that…(1–6)			
Item 1. Helps me to find my way and orient myself.	.818	.096	
Item 2. Helps me to feel safe and secure for my work tasks.	.861	.227	
Item 3. Helps me to perform my work tasks as good as possible and without difficulties.	.823	.247	
Item 4. Helps me to create integrity/privacy for the patient.	.729	.341	
Item 5. Helps me to be in control and have choices for my work.	.781	.387	
Item 6. Gives me the opportunity to get the right light for my work tasks.	.801	.254	
coloring that…(7–11)			
Item 7. Helps me to find my way and orient myself.	.238	.848	
Item 8. Helps me to feel safe and secure for my work tasks.	.256	.910	
Item 9. Helps me to perform my work tasks as good as possible and without difficulties.	.217	.919	
Item 10. Helps me to create integrity/privacy for the patient.	.280	.866	
Item 11. Affects my job positively.	.272	.776	
Eigenvalue	6.5	1.9	
% of variance explained, %	59.2	17.1	
Cumulative percentage of variance explained = 76.3%			
Cronbach’s coefficient α = .93			

### Internal Consistency Reliability

The total Cronbach’s coefficient α is .94 for LCQ-P/F and .93 for LCQ-S. The total Cronbach’s coefficient α is .94 for LCQ-P/F; it is .90 for the Light subscale and .93 for the Color subscale. LCQ-S’s total Cronbach’s coefficient α is .93, while it is .92 for the Light subscale and .94 for the Color subscale (Table 7).

**Table 7.** Description of Reliability Figures Regarding the Light and Color Questionnaire (LCQ) With Reference to Patients/Family Members (LCQ-P/F) and Staff (LCQ-S).

Questionnaire Details	Cronbach’s Coefficient α	Corrected Item-Total Correlation (Range)	Cronbach’s α If Item Deleted (Range)
Patient/family			
Items 1, 2, 3, 5, 6 (light)	.90	.72–.81	.87–.89
Items 4, 7–11 (color)	.93	.66–.86	.91–.93
All Items 1–11	.94	.67–.83	.93–.94
Staff			
Items 1–6 (light)	.92	.70–.82	.89–.91
Items 7–11 (color)	.94	.74–.91	.92–.95
All Items 1–11	.93	.70–.82	.89–.91

## Discussion

This study presents the development and evaluation of a new self-report questionnaire instrument—the LCQ-P/F and LCQ-S—designed to assess how light and color are perceived as being supportive for patients, family members, and staff in the physical care environment of an ED. The construct validity and internal consistency reliability of the instrument were evaluated with good outcomes by analyzing 600 questionnaires completed before and after an intervention consisting of refurbishing and remodeling an ED (results of the intervention will be reported elsewhere). Since the support needs differed somewhat between patients, family members, and staff, two questionnaires were created, one for patients and family members (LCQ-P/F) and one for staff members (LCQ-S). It is possible that other items that were not included would have affected the validity and reliability of the instrument.

***This study presents the development and evaluation of a new self-report questionnaire instrument—the LCQ-P/F and LCQ-S—designed to assess how light and color are perceived as being supportive for patients, family members, and staff in the physical care environment of an ED***.

***Since the support needs differed somewhat between patients, family members, and staff, two questionnaires were created, one for patients and family members (LCQ-P/F) and one for staff members (LCQ-S)***

The results of our study thus establish the validity and reliability of the LCQ-P/F and LCQ-S and contribute to the literature by providing a means to assess the extent to which the light and color of a healthcare environment are perceived by users as being supportive. As such, the instruments could be used for general environmental audits as baseline measure before refurbishments, as an evidence-based reflection tool for critical discussions of environmental optimization, or as a research tool to generate environmental variables in future studies. Psychometric testing of the instruments in other care settings or with other samples could contribute to the instruments’ further development.

***The results of our study thus establish the validity and reliability of the LCQ-P/F and LCQ-S***.

Items on the LCQ-P/F and LCQ-S were based on existing dimensions of the PEAP (Weisman, 1994) which is to our understanding the best validated instrument focusing on supportive physical care environments for users (Elf et al., 2017) and a strength of our study. Yet a limitation with the PEAP is that it was developed and used for elderly patients and only assesses experiences with the physical setting from a manager’s perspective. In contrast to the PEAP, experiences from the users’ perspectives are in line with a person-centered approach to caregiving, and we therefore developed the LCQ as a self-report instrument.

An expert group and a staff group assessed the questionnaires’ face validity and provided feedback on content and readability. As mentioned, the LCQ-P/F and LCQ-S demonstrated high internal consistency but also a sufficient homogeneity of the instruments’ assertions (Shadish et al., 2002) indicating acceptable reliability (Rattray & Jones, 2007). To explore the construct validity of the scales and get as clear a factor solution as possible, explorative factor analysis was conducted using principal component analysis with varimax rotation and Kaiser normalization (Streiner et al., 2015). Factor analysis showed that the LCQ-P/F and LCQ-S subscales seem to measure what they intend to measure regarding light and color. In the present study, exploratory factor analysis demonstrated that light as support and color as support were two quite distinct factors resembling the underlying construct with highly satisfactory explained variance. For the LCQ-P/F, the first (strongest) extractor factor with the highest level of explained variance was *color* as support, whereas for LCQ-S, it was *light* as support. The results point to *color* as support being of greater importance to patients and family members, probably due to being in an unfamiliar environment where it is essential to find visual contrasts. Through color, humans are able to interpret what they see (Billger et al., 2017), and through contrasts, information is perceived in the environment that facilitates orientation in a new milieu (Wijk & Häggström, 2017). The results reveal that light as support was of greater importance to staff members, probably because they are in a familiar environment. Becoming habitual and comfortable in an environment, as a staff member, more light than color is needed to perform work-related tasks.

***The results point to *color* as support being of greater importance to patients and family members, probably due to being in an unfamiliar environment where it is essential to find visual contrasts. Through color, humans are able to interpret what they see***.

***. . . and through contrasts, information is perceived in the environment that facilitates orientation in a new milieu***

Item 4 (light) on LCQ-P/F, *helps me feel private*, with the maximum value for color as support, had an impact on both light and color support factors, which means that patients and family members respondents perceived Item 4 as more closely related to color than to light. Normally, when an item is a misfit, it is possible to either remove or rewrite the item. However, the research team scrutinized the data and decided, based on the high-factor loadings in Factor 1, that Item 4 belongs to color as support on the LCQ-P/F. Since color and light seem to be so closely connected to the *feeling of privacy*, especially for patients (Table 5), one possible amendment is to merge Item 4 (color as support*—*to feel private) and Item 10 (light as support*—to feel private*) and treat them as a single item, not included in the factors. To understand the complexity of these phenomena more fully, the LCQ-P/F and LCQ-S Questionnaires need further development. The instruments’ use in other units and healthcare contexts remains to be tested in future research, in line with confirmatory factor analysis.

The goal of the LCQ-P/F and LCQ-S is to increase awareness of how to develop, evaluate, and plan more secure and supportive care environments in the future with other questionnaires. The LCQ-P/F and LCQ-S may also be a contribution to filling the gap in assessing the extent to which users perceive the physical care environment as person-centered. A comprehensive caring approach includes a supportive physical care environment for patients, family members, and staff (Caspari et al., 2011; Gesler et al., 2004; Huisman et al., 2012; Ulrich et al., 2008) even if studies incorporating these views are sparse. Hence, implementing evidence-based design approaches complemented by assessments of how users perceive the physical care environment is vital (Andrews et al., 2008; Salonen et al., 2013).

***The goal of the LCQ-P/F and LCQ-S is to increase awareness of how to develop, evaluate, and plan more secure and supportive care environments in the future with other questionnaires***.

## Conclusion

The LCQ-P/F and LCQ-S self-report questionnaires were developed to evaluate the extent to which light and color are perceived as being supportive for patients, family members, and staff members. The psychometric evaluations showed satisfactory validity and reliability and support a tentative use of the new questionnaires for further research and development purposes. This may be useful for architects, administrators, and researchers of healthcare environments as a baseline measure prior to refurbishments, as an evidence-based reflection tool for critical discussions of environmental optimization, or as a research tool to generate environmental variables in future studies.

## Implications for Practice

The self-reported questionnaire LCQ was developed; hence, no suitable self-report instrument in the literature was found to evaluate the perceived support of light and color from the users’ perspective—patient, family members, and staff.

The main implications for practice are as follows:

- LCQ—used as a tool for evaluation, research, and development of existing care environments to optimize supportive light and color.
- LCQ—showed satisfactory content and internal validity and high internal consistency to support the use of the questionnaire for research and development purpose.
- LCQ—could be used with other tools to evaluate, develop, and improve the design of future healthcare environments and for implementing person-centered care.
- LCQ—could be base for policies and guidelines.
- LCQ—useful for architects, administrators, and researchers and for interdisciplinary research.
- LCQ—may generally increase the awareness of the importance of the physical care environment.
